# Using Machine Learning to Predict Depression among Adolescents Living with HIV in Uganda

**DOI:** 10.1007/s40609-026-00456-3

**Published:** 2026-03-21

**Authors:** Vicent Ssentumbwe, Francis Matovu, Phionah Namatovu, Claire Najjuuko, Samuel Kizito, Josephine Nabayinda, Portia Nartey, Flavia Namuwonge, Proscovia Nabunya, Chenyang Lu, Massy Mutumba, Fred. M Ssewamala

**Affiliations:** 1 Division of Computational and Data Science, Washington University in St. Louis, St. Louis, MO, USA; 2 International Center for Child Health And Development (ICHAD), Masaka, Uganda; 3 International Center for Child Health And Development (ICHAD), St. Louis, St. Louis, MO, USA; 4 Department of Computer Science and Engineering, Washington University in St. Louis, St. Louis, MO, USA; 5 School of Public Health Sciences, Washington University in St. Louis, St. Louis, MO, USA; 6 Silver School of Social Work, New York University, New York, USA

## Abstract

**Background:**

Adolescents living with HIV (ALWHIV) face a high prevalence of depression. In Uganda, limited access to mental health services exacerbates depression, necessitating innovative solutions for early detection and intervention. Machine learning (ML) provides a promising approach for identifying adolescents at risk of depression to facilitate early intervention. This study aims to use machine learning to predict depression among ALWHIV in Uganda.

**Methods:**

This study analyzed cross-sectional data from 833 ALWHIV aged 10–17 years in Southern Uganda. Depression was assessed using the Children’s Depression Inventory (CDI) –short version. Seven ML models, including Random Forest, Logistic Regression, Gradient Boosting, decision tree, XGBoost, Least Absolute Shrinkage and Selection Operator (LASSO), and Support Vector Machine, were evaluated using stratified 10-fold cross-validation. Model performance metrics included Area under receiver operating characteristic curve (AUROC), Area under precision-recall curve (AUPRC), accuracy, sensitivity, specificity, and F1-score. SHapley Additive exPlanations (SHAP) analysis was used to interpret feature importance.

**Results:**

The median age was 13 years, and the majority of the participants were females (55.5%). The prevalence of depression was 30.97%. The Random Forest model achieved the highest performance (AUROC = 0.79, AUPRC = 0.66). Key predictors of depression included hopelessness, HIV stigma, shame, self-esteem, teacher support, and caregiver support. SHAP analysis highlighted psychosocial challenges as primary predictors of depression, emphasizing the need for psychosocial-based interventions.

**Conclusion:**

Our study results demonstrated good performance of ML in predicting depression among ALWHIV. However, we acknowledge that the absence of external validation limits the generalizability of these findings. Future research should seek to validate these models in independent cohorts of ALWHIV to confirm their robustness and applicability across different settings.

## Introduction

Globally, an estimated 39.9 million people are living with HIV (UNAIDS, 2023), including approximately 2.1 million adolescents aged 10–19 years (UNAIDS, 2024b). Sub-Saharan Africa (SSA) remains the most affected region, hosting 67% of all people living with HIV. In 2023, Eastern and Southern Africa alone accounted for an estimated 20.8 million individuals living with HIV (UNAIDS, 2024a). Uganda, one of the SSA countries hardest hit by the epidemic, has over 72,000 children aged 0–14 years living with HIV (Vreeman et al. 2017).

Beyond the physical health challenges associated with HIV, mental health concerns are a growing and serious issue among adolescents living with HIV (ALWHIV) in SSA (Vreeman et al. 2017). Globally, 1 in 7 adolescents aged 10–19 years is estimated to experience a mental health condition, with SSA reporting the highest burden (Vreeman et al. 2017). Studies have reported depression prevalence ranging from 16% to 40.8% among young people, with an even higher prevalence (44%) among ALWHIV compared to their HIV-negative peers (Too et al. 2021). Depression among ALWHIV is consistently associated with poor quality of life, increased stigma, and heightened suicidal ideation (Mudra Rakshasa-Loots et al. 2022, Brown et al. 2000). It adversely impacts health outcomes, including poor adherence to antiretroviral therapy (ART), leading to increased viral load and worsened HIV prognoses (Mudra Rakshasa-Loots et al. 2022, Brown et al. 2000).

ALWHIV in schools face heightened risks of depression and other mental health issues due to stigma and social isolation in the school environment, which can worsen HIV outcomes (Kimera et al. 2019, Kimera et al. 2020). Bullying within school settings has been identified as a critical psychosocial stressor that intensifies stigma, undermines social support, and increases vulnerability to depression among ALWHIV (Ashaba et al. 2018). Additionally, school-going ALWHIV report fears and negative self-perceptions, as well as difficulties managing antiretroviral therapy (ART) due to concerns about disclosure of their HIV status to peers (Kimera et al. 2020, Mutumba et al. 2015). Together, these intersecting challenges exacerbate emotional distress and contribute to elevated levels of depression in this population. In Uganda, these challenges are compounded by limited access to mental health care, due to a substantial treatment gap (Iversen et al., 2021); hence, the diagnosis and treatment of depression among ALWHIV become a critical public health concern. The country faces a shortage of mental health professionals, limited integration of mental health services into routine HIV care, and pervasive stigma surrounding both mental health and HIV (Iversen et al., 2021). These barriers hinder timely diagnosis and effective treatment, leading to unaddressed mental health needs that worsen HIV treatment outcomes. Addressing this gap requires innovative approaches that can supplement resource-limited health systems and improve access to mental health care.

Machine learning (ML) techniques offer a promising solution for predicting depression and other mental health challenges. ML models can analyze complex, high-dimensional datasets to identify individuals at risk of depression with greater precision than traditional statistical methods (Rajula et al. 2020). By enabling early detection, ML can facilitate timely interventions, potentially mitigating longterm adverse outcomes and improving the quality of life for ALWHIV (Cho et al. 2019, Dobias et al. 2022, Chung and Teo 2022). However, early and accurate identification of depression among ALWHIV is critical but insufficient in Uganda. To be impactful, detection must be linked with scalable and contextually appropriate interventions, such as school-based psychosocial programs, integration of mental health into HIV care platforms, and the use of lay counselors or peer supporters to deliver brief evidence-based therapies. This highlights the importance of developing approaches that not only predict individuals at greatest risk of depression but also inform strategies to connect them to feasible care pathways.

Machine learning (ML) has been widely used to predict depression and identify risk factors in various populations, but most studies have been conducted in high-income settings and focus on general adolescent or adult groups (Qasrawi et al. 2022, Yoo et al. 2024, Marathe et al. 2022). In Uganda, Brathwaite et al. (2021) developed a model to predict mental health functioning among adolescents orphaned due to HIV, but the study relied solely on LASSO regression, limiting the exploration of alternative, potentially more robust algorithms (Brathwaite et al. 2021). While ML techniques have demonstrated significant potential in predicting mental health problems, including depression and informing early interventions, their application remains limited in SSA, particularly among ALWHIV. Given the similar mental health risks observed globally, leveraging ML for depression prediction among ALWHIV could provide contextually relevant, data-driven solutions for mental health interventions. To address this gap, the current study leverages state-of-the-art ML techniques to predict depression among school-going ALWHIV, aiming to provide data-driven solutions for early detection of depression.

### Theoretical Framework

This study is guided by Bronfenbrenner’s socioecological model (SEM) (Bronfenbrenner 1977). The model considers the complex interplay between individuals, relationships, communities, and societies to understand how health and behaviors are affected (Bronfenbrenner 1977). The model posits that individuals and their environment influence each other, and that addressing behaviors at multiple levels is most effective. This model is particularly relevant for understanding depression among ALWHIV in Uganda, as it captures the complex factors that contribute to mental health challenges in this population. At the individual level, the SEM highlights the role of personal attributes in shaping health outcomes. For ALWHIV, stigma, shame, and hopelessness are central to their experience and are documented as major contributors to depression (Katz et al. 2013). For example, the stigma associated with HIV can lead to internalized negative beliefs, resulting in feelings of worthlessness and despair, which are strongly linked to depression (Rueda et al. 2016, Ashaba et al. 2019). At the relationship level, the SEM emphasizes the influence of relationships and social networks such as family, caregivers, and teachers. ALWHIV are particularly dependent on supportive relationships to cope with the challenges of living with HIV. Conversely, strained family relations or lack of social support can exacerbate feelings of isolation, increasing vulnerability to depression (Lee et al. 2007). At the community level, SEM focuses on broader systemic and structural factors such as access to healthcare for ALWHIV, barriers to healthcare, including stigma in healthcare settings, limited mental health resources, financial constraints that discourage help-seeking behaviors, and perpetuate feelings of isolation, which is linked to depression (Mugisha et al. 2020, Betancourt et al. 2013). With this framework, we believe that the interplay of these factors is influential in predicting depression for ALWHIV.

## Methods

### Study Design and Population

We utilized cross-sectional data from a three-arm cluster-randomized controlled trial (M-Suubi), designed to reduce the impact of HIV stigma on HIV treatment outcomes among ALWHIV in Ugandan schools (Mutumba et al. 2022). A total of 833 ALWHIV, with their caregivers, were enrolled in the study. ALWHIV were eligible to participate if they were: (1) HIV positive, confirmed by a medical report and had disclosed to their HIV status; (2) prescribed antiretroviral therapy (ART); (3) living within a family; and (4) aged 10 to 17 years and enrolled in a primary or secondary school. ALWHIV were recruited from 42 clinics across five geopolitical districts in southwestern Uganda. Details on participant recruitment are published in the study protocol (Mutumba et al. 2022).

### Ethical Considerations and Informed Consent

The research staff obtained written informed consent and assent from the adult caregivers and children, respectively, before study enrollment. The consenting process for parents/caregivers and adolescents was performed separately to avoid coercion. During the meeting, caregivers and adolescents were informed verbally and in writing about the purpose of the study, voluntary participation, extent of their participation, risks and benefits, as well as protection and confidentiality issues. Both consent and assent forms were translated into Luganda (the most widely spoken local language in the study region) and back-translated to English to ensure consistency. Prior to data collection, the study team received training on Good Clinical Practice and completed Collaborative Institutional Training Initiative to ensure human subject protection. Detailed information on the participants’ recruitment and selection process, power analysis, as well as the intervention is described in the study protocol (Mutumba et al. 2022).

All study procedures were approved by the institutional review boards (IRBs) at Washington University in St. Louis (IRB ID 202201128) and the University of Michigan (HUM00211945) and by the in-country local IRBs in Uganda: Uganda Virus Research Institute (GC/127/867) and Uganda National Council of Science and Technology (SS1166ES). The study was registered with ClinicalTrials.gov (NCT05307250).

### Data collection and Measures

Data were collected using a ~ 90-minute interviewer-administered survey. Interviews were conducted in Luganda, the local language, by trained Ugandan interviewers. All measures utilized in this study were tested in our previous studies in Uganda among children and adolescents affected by HIV/AIDS in the study area (Ssewamala et al., 2009; Sse- wamala et al., 2016; Ssewamala et al., 2012). We tested the reliability of scale items to ensure internal consistency. We have presented a description and Cronbach’s alpha for each measure in the supplementary file (Table 1).

## Measures

### Outcome Variable

The outcome variable, depression, was assessed using the short version of the Child Depression Index (CDI) (Kovacs 1992, Kovacs 2015). Adolescents were asked to describe their feelings over the past two weeks using a series of statements that measure depressive symptoms such as “sadness,” “pessimism,” “self-deprecation,” “self-hate,” “crying spells,” “irritability,” “negative self-image,” “loneliness,” “lack of friends,” and “feeling unloved.” The CDI has been widely used in pediatric and adolescent populations as a screening tool in diverse cultural contexts and has demonstrated acceptable validity and acceptable reliability with a meta-analytic Cronbach’s alpha of approximately 0.77 (Sun & Wang, 2015). The measure has also been used in studies involving adolescents in sub-Saharan Africa, including Uganda and Rwanda (Mutumba et al. 2014, Binagwaho et al. 2016). Each item on the CDI included three response options, with scores of 0 indicating no symptom, 1 indicating a mild or probable symptom, and 2 indicating a definite symptom. Five items in the inverse direction were reverse-coded to create a summated score. Higher scores indicated higher levels of depressive symptoms, with a theoretical score range of 0 to 20. To categorize participants, scores were dichotomized: a score of 3 or above indicated depressive symptomatology (categorized as “depressed”), while a score below 3 indicated the absence of depressive symptoms (categorized as “non-depressed”) (Allgaier et al., 2012).

Guided by the SEM, we purposively selected features that are associated with depression among ALWHIV at different levels. At the individual level, we include demographic characteristics like gender and age, clinical records e.g., HIV viral load, self-reported ART medication adherence). At the interpersonal level, we included family factors, for example, family income, family cohesion, care, and relationships, and social support networks. At the community level, barriers to medical care were included. A detailed description of predictors in this sample is given in the supplementary material [Supplementary Table 1].

### Data Processing

Prior to analysis, we performed rigorous preprocessing of the dataset to ensure its cleanliness. Three variables had missing data: self-esteem (1.56%), food insecurity (0.12%), and viral load (0.24%). Given the low level of missingness (< 2%), we used listwise deletion to ensure a complete case dataset suitable for ML applications. Categorical predictors were transformed using one-hot encoding, a method that converts each categorical variable into multiple binary (0 or 1) variables representing distinct categories, and continuous variables were standardized (Zheng and Amanda 2018).

### Feature Selection

Given the high dimensionality of the dataset, feature selection was performed to reduce model complexity, thereby mitigating the risk of overfitting and enhancing interpretability. We utilized the Least Absolute Shrinkage and Selection Operator (LASSO) for this purpose (Tibshirani 1996). LASSO is a regularization technique that applies a penalty to the absolute size of the regression coefficients, effectively shrinking the coefficients of less contributive variables to zero. By excluding these variables from the model, LASSO retains only the most predictive features, ensuring a parsimonious and robust model for predicting depression.

### Model Training and Validation

To predict depression among adolescents, we employed a diverse set of machine learning algorithms, including Random Forest (RF), Decision Trees (DT), Gradient Boosting (GB), Extreme Gradient Boosting (XGBoost), Support Vector Machine (SVM), LASSO, and Logistic Regression (LR) (Cho et al. 2019; Kotsiantis 2007; Babcock University et al. 2017).

We implemented stratified 10-fold cross-validation to ensure robust and unbiased model evaluation. In this framework, the dataset was split into 90% for training and 10% for testing across 10 iterations. Stratification allowed us to maintain the same distribution of the target variable in the train and test sets across folds. This is particularly critical given the imbalanced nature of our dataset (31% depressed cases). Model performance was evaluated using the following metrics: (1) Specificity which is a proportion of actual negatives (non-depressed), which got predicted as nondepressed (true negative) (Sokolova & Lapalme, 2009); (2) Sensitivity; a measure of a proportion of actual positives (depressed) that got predicted as depressed (true positives) (Sokolova & Lapalme, 2009); (3) Accuracy; that measures how correctly a model predicts an outcome (Sokolova & Lapalme, 2009; Hossin & Sulaiman, 2015); (4) Precision which measures the positive cases that are correctly predicted from the total predicted cases in a positive class; (5) F1 score - the harmonic mean between sensitivity and precision values (Hossin & Sulaiman, 2015); (6) Area Under the Receiver Operating Characteristic Curve (AUROC); a measure of discriminatory capacity of classification models (It takes into account sensitivity and specificity) (Sokolova & Lapalme 2009). AUROC measures the model’s ability to distinguish between classes across all classification thresholds (Fawcett, 2006; Bradley, 1997), and (7) Area Under the Precision-Recall Curve (AUPRC) that evaluates the overall model performance based on precision and recall (Hossin & Sulaiman 2015, Sokolova & Lapalme 2009). In addition, we fixed the sensitivity (recall) to a high value of 0.80 during model optimization and evaluated the model specificity, accuracy, and F1-score. This was done to prioritize the identification of adolescents at high likelihood of being depressed to ensure high-risk individuals are identified for potential interventions. This rigorous training and testing process ensured that our models were robust and aligned with the study’s goal of developing an accurate and sensitive tool for predicting depression among ALWHIV.

### Feature Importance Analysis

We employed SHapley Additive exPlanations (SHAP) to gain a better understanding of the contributions of individual predictors to the model’s predictions (Lundberg & Lee, 2017). SHAP is a game-theoretic approach to explain the output of machine learning models by attributing importance scores to each feature based on its contribution to the model’s predictions (Lundberg & Lee, 2017). SHAP values quantify how much each feature increases or decreases the predicted outcome for a specific observation. We generated the SHAP dot-plot to show how the top features contribute to the predictions made by the model. Additionally, we plotted SHAP waterfall plots to further examine the differences in the feature contributions to model prediction for a few selected individuals.

### Sensitivity Analysis

To ensure the robustness of our conclusions, we conducted sensitivity analyses by applying oversampling techniques, specifically Synthetic Minority Over-sampling Technique (SMOTE) and Adaptive Synthetic Sampling (ADASYN). These methods were used to address class imbalance and assess their impact on model performance. We retrained all seven algorithms and evaluated their performance using the previously defined metrics to determine whether oversampling improved model performances. Additionally, we conducted a sensitivity analysis to assess the robustness of our findings on viral load suppression. In the primary analysis, viral suppression was defined as a viral load of < 1,000 copies/mL, consistent with the World Health Organization (WHO) guidelines (WHO, 2023). For the sensitivity analysis, we applied a more stringent threshold of < 200 copies/mL, as recommended by the Uganda Ministry of Health in the most recent Consolidated Guidelines for the Prevention and Treatment of HIV (Uganda Ministry of Health, 2022). We then evaluated whether this categorization affected the model’s predictive performance.

All data analyses were conducted using Python 3.7 with pandas (McKinney, 2011), scikit-learn (Pedregosa et al. 2011), XGBoost (Chen et al. 2016), and SHAP (Lundberg & Lee, 2017) libraries.

## Results

Participants’ characteristics are shown in Table 1. The median age was 13 years (IQR: 12–13). Slightly more than half of the participants were female (55.9%). About 38% of the participants were orphans (i.e., had lost one or both parents), and 55.1% reported biological parents as their primary caregivers. The median asset ownership score was 11 (IQR: 9–13). Close to one-third of participants (30.97%) were classified as depressed.

### Identification of the Most Relevant Predictors

Following the LASSO-based feature selection, 12 out of 31 predictors were maintained for predicting depression. These included: HIV stigma, hopelessness, Child caregiver support, teacher social support, food insecurity, adherence self-efficacy, HIV shame, stigma by association at school, self-esteem, self-concept, self-reported poor ART-adherence, and orphanhood status. The LASSO coefficients are shown in the supplementary material (Supplementary Table 2).

### Model Evaluation

Out of seven models, the RF classifier achieved the highest predictive performance with an AUROC of 0.79 (0.03) and AUPRC of 0.62 (0.07). At a 0.80 sensitivity threshold, RF obtained the highest specificity of 0.66 (0.07); accuracy of 0.71 (0.05); precision of 0.52 (0.06), and F1-score of 0.64 (0.04). (Table 2)

### Feature Importance

**Fig. 1:**
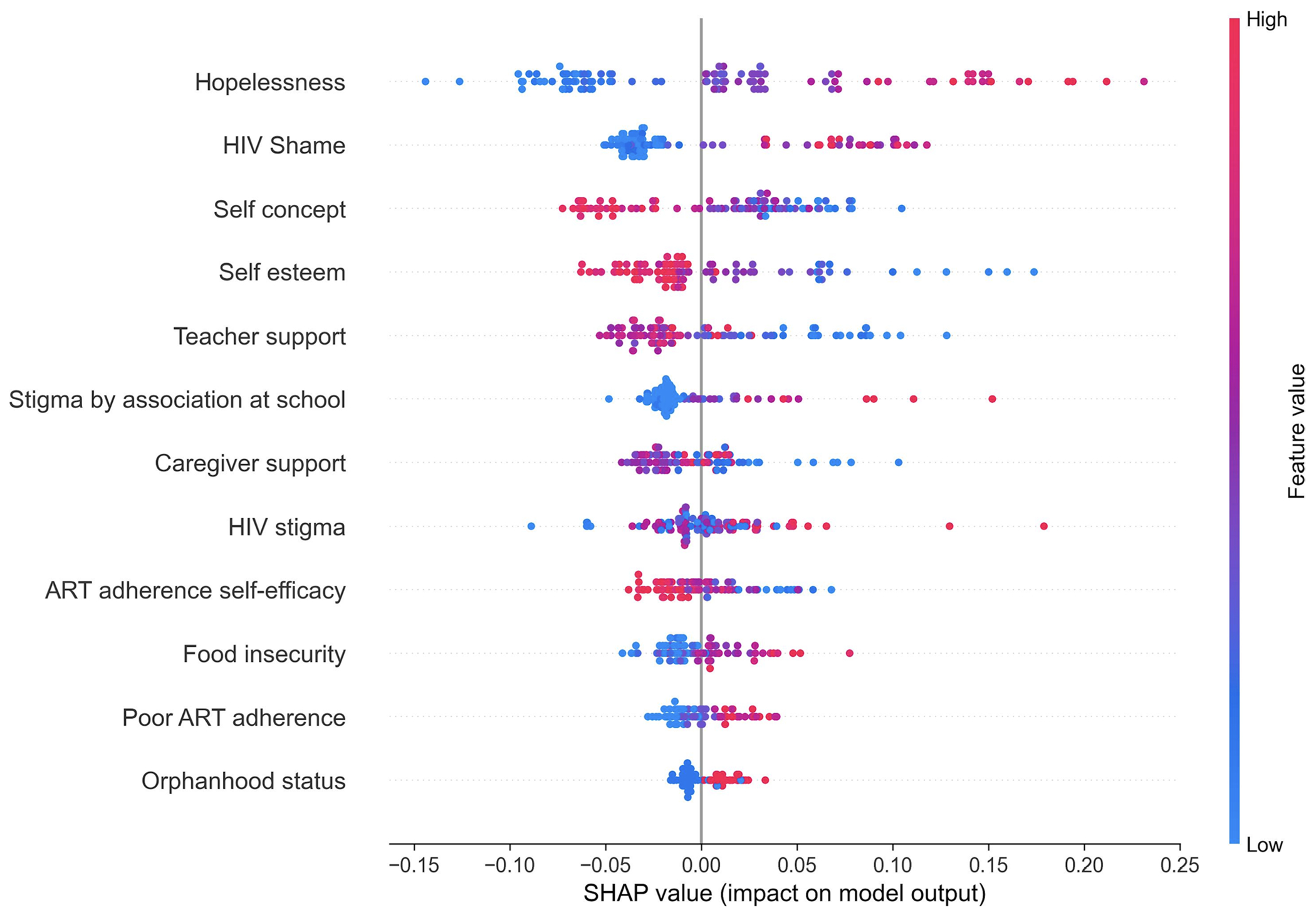
SHAP summary dot plot. The y-axis shows the top 12 most contributive features to the model’s predictions; Features at the top are more important (higher contribution) to the model compared to those at the bottom. The x-axis (SHAP Value) represents the impact of a feature on the model’s output (prediction). Positive SHAP values (to the right of the central gray axis) push the model toward predicting “depressed” and negative SHAP values (to the left of the gray central axis) push the model toward predicting “non-depressed.” Dots are colored according to the feature value, with higher values in red and lower values in blue

**Fig. 2:**
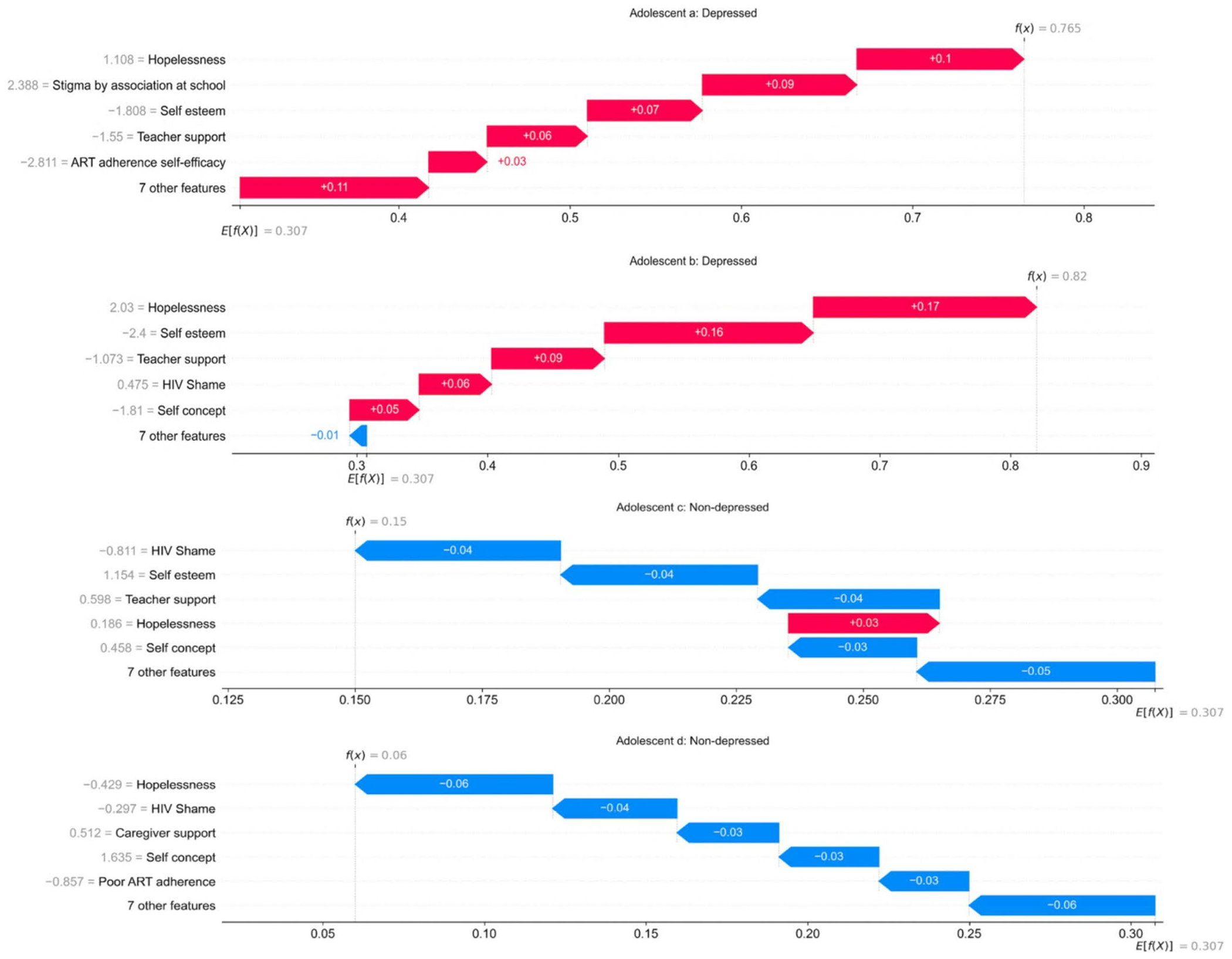
Feature contributions to model predictions for four adolescents; two classified as being depressed and two classified as not being depressed

The SHAP dot-plot in Fig. 1 shows the relative contributions of the best features to the RF model’s predictions, from the most (top) to the least (bottom) contributive features. Figure 1 summarizes the impact of explanatory features on the model output. Features that increase or decrease the likelihood of depression are coded in red and blue, respectively. From the SHAP plot, hopelessness was the highest predictor of depression, whereby high values (red) strongly increase the likelihood of predicting an individual as depressed. High levels of HIV shame were also highly predictive of depression. Lower values of self-concept, self-esteem, social support from teachers, caregiver support, ART adherence self-efficacy (blue) increase the likelihood of predicting an individual as depressed. Additionally, higher values of stigma by association at school, food insecurity, poor ART adherence, and being an orphan (red) increase the likelihood of an individual being depressed; however, they have less influence on the model’s predictions (Fig. 2).

The SHAP waterfall-plots above show a sample of individual predictions for adolescents classified as being depressed and those not depressed, while highlighting the most contributive features for each prediction. The first adolescent (a) has a higher predicted probability of being depressed (0.77) due to the strong positive contribution of hopelessness, stigma by association at school, and lower values of self-esteem and teacher support. The second adolescent (b) has a higher predicted probability of being depressed (0.82) due to the strong positive contributions of hopelessness, HIV shame, and lower values of selfesteem, teacher support, and self-concept. Adolescent (c) has a lower predicted probability of being depressed (0.15), in other words, a high probability of being non-depressed (0.85) due to the strong protective contributions of low levels of HIV Shame, and higher levels of self-esteem, and teacher support. The slightly high levels of hopelessness push the prediction to the right; however, its contribution is not sufficient to counteract the strong contribution of other factors. Adolescent (d) has a very low predicted probability of being depressed (0.06) or a higher probability of being non-depressed (0.94) driven by the strong protective contributions from low levels of hopelessness, HIV shame, poor ART adherence, and higher values of caregiver support and self-concept.

### Sensitivity Analysis

Results from the sensitivity analyses addressing class imbalances indicated no improvement in the model performances, therefore, we maintained our original model. Using SMOTE, results show that out of seven models, SVM achieved the highest predictive performance with an AUROC of 0.77 (0.05) and; AUPRC of 0.61 (0.06). At a 0.80 sensitivity threshold, a specificity of 0.58 (0.09) was obtained; accuracy of 0.65 (0.06); precision of 0.47 (0.06), and F1-score of 0.60 (0.04). Using ADASYN balancing technique, logistic regression was the best classifier with an AUROC of 0.77 (0.04), AUPRC of 0.60 (0.06) and at 0.80 sensitivity threshold, it obtained a specificity of 0.42 (0.16); accuracy of 0.59 (0.06); precision of 0.43 (0.04) and F1-score of 0.58 (0.03) [supplementary Tables 3 and 4].

Additionally, sensitivity analysis using a lower viral load suppression threshold of < 200 copies/mL, as recommended by the Uganda Consolidated HIV Guidelines, revealed no significant changes in predictive performance. Viral load was not retained among the top predictive features in LASSO model. The results were consistent with those obtained using the < 1,000 copies/mL threshold recommended by the WHO; therefore, the primary analyses were maintained using the < 1,000 copies/mL definition of viral suppression.

## Discussion

In this study, we applied machine learning techniques to predict depression among ALWHIV in Uganda and examined the factors associated with depression. Results indicate the RF classifier as the most effective model for predicting depression in our sample. It achieved the highest AUROC (0.79) and AUPRC (0.62) compared to other ML models. SHAP analysis identified hopelessness, HIV shame, self-concept, and teacher support as the most significant contributors to the model’s predictions.

Our findings demonstrate that machine learning techniques are effective in identifying ALWHIV who are at an elevated likelihood of depression. RF outperformed other ML models due to its ability to handle the heterogeneity of predictors of depression by constructing numerous decision trees and combining them to get a more accurate and reliable prediction while avoiding overfitting (Cho et al. 2019). This finding aligns with prior research that also identified RF as the best model for similar predictive tasks (Priya et al. 2020, Sau and Bhakta 2017, Haque et al. 2021), while other studies have shown that other ML techniques like DT (Colledani et al., 2023) and SVM (Qasrawi et al. 2022) also perform well in predicting depression, reinforcing the ability of ML methods in handling complex or high-dimensional data compared to traditional statistical approaches (Lee et al. 2018, Kessler et al. 2016). Other studies have demonstrated the effectiveness of more advanced ML methods, such as neural networks (Baek and Chung 2020) and multi-task learning (Dai et al., 2022), in predicting depression. However, we did not implement these approaches in our study due to data limitations, as their application could lead to overfitting. Moreover, studies using survey datasets to predict mental health outcomes, including depression, reported AUROCs ranging from 0.556 to 0.788, which are comparable to our results (Yoo et al. 2024, Hawes et al. 2023, Brathwaite et al. 2020).

The analysis identified 12 key predictive factors from a list of 32 spanning from individual, interpersonal, and community levels as guided by the SEM. The SEM framework provides a deeper understanding of the diverse determinants associated with depression, emphasizing the need for multi-level interventions to address depression among ALWHIV (Buzi et al. 2015, Gu et al. 2014).

Among individual-level predictors, hopelessness and HIV shame emerged as particularly important factors associated with depression. These findings may be explained by the fact that hopelessness and HIV shame can impact an individual’s cognitive, emotional, and social function (Li et al. 2009, Nabunya et al. 2023). Hopelessness is characterized by negative expectations about the future and a perceived inability to influence health outcomes, leading to feelings of helplessness and despair (Beck 1967). Moreover, HIV shame often stems from societal stigma, internalized negative beliefs, and fears of rejection or discrimination, which can erode self-worth and exacerbate feelings of isolation (Nabunya et al. 2023). These psychosocial challenges create a cycle of negative affect, social withdrawal, and impaired resilience, which is especially pronounced during the sensitive developmental stage of adolescence and in resource-limited settings where support systems and mental health services are often inadequate. Additionally, self-esteem and self-concept were found to be vital in buffering against depression. High self-esteem fosters a positive self-image and resilience, enabling individuals to navigate the stigma and challenges associated with their condition. Similarly, a strong self-concept enhances confidence and self-worth, reducing susceptibility to negative thoughts and emotions. Collectively, these factors underscore the importance of social and psychological interventions in creating a supportive ecosystem that promotes mental well-being and mitigates its adverse effects.

Among interpersonal factors, high levels of social support from teachers and caregivers were associated with a lower likelihood of an individual being predicted to have depression. Social support from teachers provides a sense of belonging and validation, which can counteract feelings of isolation and marginalization, mainly at school (Nyoni et al. 2019, Nabunya et al., 2020, Peñate et al. 2020). Similarly, family relationships and support provide emotional security and practical support, reinforcing a stable environment where adolescents feel valued and understood (Pössel et al. 2018). HIV stigma, mainly externalized stigma which is thought to stem from individuals’ community surrounding, was also associated with depression. HIV stigma among ALWHIV can lead to feelings of isolation and low self-esteem, causing negative perceptions about themselves, which can significantly impact their depression and overall mental health (Nabunya et al. 2023).

The individual predictions further emphasize the distinct influence of various factors in predicting depression among ALWHIV (Figs. 3 and 4). The results highlight that certain factors like HIV stigma, shame, and hopelessness, consistently increase the likelihood of depression, while factors like support from caregivers and teachers contribute to lower depression probabilities. The findings highlight the need for individualized interventions to address the unique psychological and social challenges faced by ALWHIV. Given the variability in depression risk factors, a tailored approach is essential rather than a one-size-fits-all strategy. For those at a higher likelihood of depression, interventions should focus on strengthening emotional resilience, fostering supportive relationships, and implementing school-based stigma reduction programs. Providing access to mental health counseling, such as cognitive-behavioral therapy (CBT), can help adolescents develop coping mechanisms to manage feelings of hopelessness, shame, and stigma (Nabunya et al., 2024; Young, 2018). Also, interventions should aim at reinforcing adolescents’ self-esteem, self-concept, and strong social support networks. School-based mental wellbeing programs, mentorship initiatives, and peer-support groups can help sustain these protective effects. By adopting a personalized and data-driven approach, policymakers and practitioners can design more effective mental health programs that foster mental well-being among ALWHIV.

It is also important to acknowledge that the dataset used in this study was derived from a research trial, which provided detailed psychosocial, behavioral, and clinical variables. Such rich and complex datasets are rarely available in routine health system settings in many LMICs, where data infrastructure remains limited. This restricts the immediate applicability of our models for real-world screening. Nonetheless, as digital health systems expand and routine monitoring improves, the feasibility of applying ML models may increase, particularly if integrated into routine HIV care platforms.

## Limitations

While the study provides valuable findings, they should be interpreted with caution due to several limitations. First, the study’s cross-sectional design limits causal inference, as it does not establish temporal relationships between predictors and depression. Data collection for this study is still ongoing, and we look forward to analyzing longitudinal data once data collection for all study waves is complete to explore causal pathways and better understand the dynamics of depression among ALWHIV over time. Second, the reliance on self-reported data introduces potential biases, including social desirability bias, particularly for sensitive topics such as depression. This may lead to underreporting or overreporting of certain behaviors, resulting in potential misestimation of depression levels. The use of objective measures such as biomarkers may help enhance the accuracy and robustness of depression assessment. Third, the study focused exclusively on school-going adolescents, excluding out-of-school adolescents who may experience a heightened risk of depression due to factors such as poverty, lack of social support, and limited access to healthcare. Finally, the study findings were not validated using external datasets, limiting the generalizability of the results.

### Implications and Recommendations

This study emphasizes the potential of integrating machine learning techniques into adolescent HIV care for early identification and prevention of depression. The high AUROC and AUPRC achieved by the RF model highlight its practical utility in identifying at-risk adolescents who might otherwise be missed by traditional statistical approaches such as logistic regression. The identified predictors, including hopelessness, HIV stigma, and shame, offer an actionable need for designing targeted interventions at the individual, interpersonal, and community levels. By leveraging SHAP analysis, this study enhances interpretability, providing clinicians and policymakers with a clear framework to prioritize psychosocial and resilience-based strategies.

Building on the identified best predictors of depression, targeted interventions should be prioritized to address the specific challenges faced by ALWHIV. These include implementing CBT programs focused on alleviating hopelessness, as well as stigma reduction initiatives integrated within HIV care settings. Such interventions should be designed to enhance resilience, self-esteem, and social support. Policymakers should integrate machine learning-based mental health screening tools into routine adolescent HIV care. However, the widespread implementation of such tools in low-resource settings like Uganda remains limited by data infrastructure and workforce constraints. These tools can nonetheless provide insights to guide the design of simplified, context-appropriate screening approaches that facilitate the early identification of at-risk individuals, enabling timely interventions. We acknowledge that some of the strongest predictors identified in this study, such as HIV-related stigma, shame, and hopelessness, are not routinely captured in many existing clinical monitoring systems in Uganda. However, their inclusion highlights the multidimensional drivers of depression among ALWHIV and identifies key psychosocial domains that warrant consideration in the prediction tools of depression. Future research should refine and simplify predictive models to enhance feasibility in routine HIV care settings by using well-validated, shorter psychosocial measures that can be easily administered in busy adolescent HIV clinics in LMICs.

Additionally, healthcare workers should be trained not only to interpret machine learning model outputs but also to translate the results into actionable and individualized care plans. Expanding these efforts across various healthcare and community settings will help address the mental health needs of ALWHIV more comprehensively, particularly in resource-constrained environments like Uganda. Future studies should focus on refining these tools for broader applicability and validating their effectiveness in diverse populations. Importantly, efforts to improve identification must be paired with feasible, scalable, and contextually appropriate interventions—such as school-based psychosocial support, integration of mental health into HIV care, and peer- or lay-counselor–delivered therapies—so that early detection translates into meaningful support for adolescents’ mental health.

## Conclusion

This study demonstrates the effectiveness of machine learning techniques, particularly the Random Forest classifier, in predicting depression among ALWHIV in Uganda. By leveraging SHAP analysis, the study provides an understanding of the key factors influencing mental health outcomes. These findings provide preliminary evidence that ML approaches can help identify adolescents who may be at increased risk of depression and underscore the importance of early detection and multi-level interventions targeting at addressing hopelessness, HIV stigma, and other psychosocial challenges, while strengthening self-esteem, self-concept, and social support.

However, our models have not been externally validated, and their generalizability beyond this cohort remains uncertain. The results should therefore be interpreted as exploratory and hypothesis-generating. Future research should validate these models in independent samples and diverse contexts to establish their robustness and applicability.

## Supplementary Material

Supplement Table 1

The online version contains supplementary material available at https://doi.org/10.1007/s40609-026-00456-3.

## Figures and Tables

**Table 1:** Participant characteristics

Variable	Total sample *N* = 833
Age (median (IQR))	13 (12–15)
**Gender**	
Male	367 (44.06)
Female	466 (55.94)
**Orphanhood status**	
Double orphan	69 (8.28)
Single orphan	251 (30.13)
Non-orphan	513 (61.58)
**Primary caregiver**	
Biological parent	459 (55.10)
Grandparent	233 (27.97)
Other parent	141 (16.93)
Asset ownership (median (IQR))	11 (9–13)
**Depression**	
Yes	258 (30.97)
No	575 (69.03)

**Table 2:** Predictive performance of different ML models

Classifier	AUROC (mean (SD)	AUPRC (mean (SD)	Accuracy (mean (SD)	Precision (mean (SD)	Sensitivity (mean (SD)	Specificity (mean (SD)	F1-score (mean (SD)
**Random Forest**	**0.79 (0.03)**	**0.62 (0.07)**	**0.71 (0.05)**	**0.52 (0.06)**	**0.82 (0.01)**	**0.66 (0.07)**	**0.64 (0.04)**
Logistic Regression	0.77 (0.04)	0.60 (0.07)	0.67 (0.07)	0.49 (0.07)	0.82 (0.01)	0.61 (0.11)	0.61 (0.05)
SVM	0.76 (0.04)	0.59 (0.06)	0.65 (0.08)	0.48 (0.07)	0.82 (0.01)	0.58 (0.12)	0.60 (0.05)
Gradient Boosting	0.78 (0.03)	0.63 (0.07)	0.68 (0.05)	0.50 (0.05)	0.82 (0.01)	0.62 (0.07)	0.62 (0.04)
XGBoost	0.78 (0.03)	0.61 (0.06)	0.69 (0.05)	0.50 (0.05)	0.82 (0.02)	0.63 (0.07)	0.62 (0.03)
LASSO	0.77 (0.04)	0.60 (0.08)	0.67 (0.07)	0.49 (0.08)	0.82 (0.02)	0.60 (0.11)	0.61 (0.06)
Decision Tree	0.71 (0.03)	0.51 (0.07)	0.53 (0.11)	0.39 (0.05)	0.90 (0.05)	0.36 (0.18)	0.54 (0.04)

## Data Availability

The data used for the analysis can be availed upon submitting a reasonable request to the corresponding author. The code used for the analysis is available upon a reasonable request.
