# Supplementary material for: Using Machine Learning to Predict Depression among Adolescents Living with HIV in Uganda
Source: Glob Soc Welf. Author manuscript; Available in PMC 2026 Apr 22. (PMC13099073; doi:10.1007/s40609-026-00456-3)
Supplement: Supplement Table 1 [file NIHMS2159172-supplement-Supplement_Table_1.docx]

**Supplementary Table 1: Variable description and bivariate test showing the association between model predictors and the outcome.**

| **Variable label** | **Variable type** | **Description** | **Chisquare/ ttest** | **p-value** |
| --- | --- | --- | --- | --- |
| Gender | Categorical | Two categories coded as 1= male and 2= female | 0.06 | 0.801 |
| Age | Continuous | Participant's age in years ranging from 10 to 17 | 3.77 | <0.001 |
| Orphanhood status | Categorical | Two categories coded as 0= double-orphan, 1= Single-orphan, and 2= non-orphan | 5.26 | 0.022 |
| Family cohesion | Continuous | Family cohesion was measured by a 7-item scale adapted from the Family Environment Scale^1^ and the Family Assessment Measure.^2^ The scale assesses family members’ commitment and support for each other with items were rated on a 5-point Likert scale (1=never occurs to 5=always occurs). The theoretical scores range from 7 to 35 with high scores indicating high levels of family cohesion. Actual scores range from 8 to 35 with, Cronbach’s alpha of 0.73. | 4.94 | <0.001 |
| Child-caregiver communication | Continuous | Respondents were asked to indicate how often they discussed 11 specific topics with their caregiver. Responses were rated on a 5-point Likert scale, with 1=*never*, 2=*sometimes*, 3=*about half of the time*, 4=*most of the time*, and *always*. The theoretical range for this scale is 11-55, with higher scores indicating high communication frequency levels. The actual scores ranged from 11 to 55, with Cronbach’s alpha of 0.81 | 2.51 | 0.012 |
| Perceived Child-Caregiver Support | Continuous | Respondents were asked to rate the adults they live with, on a 17-item scale. Responses were rated on a 5-point Likert scale, with 1= *never*, 2 = *sometimes,* 3=*about half of the time*, 4=*most of the time*, and 5= *always.* Items were adapted from the social support scale which demonstrated excellent internal consistency (cronbach’s alpha: 0.82-0.90).^3^ Items in the inverse direction were reverse-coded to create summated scores. The theoretical range for this scale is 17 to 85, with high summated scores indicating high levels of perceived support from caregivers. The actual scores ranged from 17 to 81, with Cronbach’s alpha of 0.81 | 7.08 | <0.001 |
| Social support from guardian | Continuous (6 items) | Social support was measured using 30-items (Cronbach’s alpha=0.84) from the Friendship Qualities Scale.^4^ The scale assesses the impressions of the quality of children’s friendships and relationships with their guardians, classmates, teachers and peers. Respondents were asked to rate how each statement applied to them.^4^ All subscales manifested acceptable reliability with Cronbach’s alpha coefficinets between 0.71 and 0.86.^4^ The scale also exhibited good validity and reliability among adolescents in Turkey (Cronbach’s alpha coeefient between 0.66 to 0.86).^5^ Responses were rated on a 5- point Likert scale, with 1=*never*, 2=*sometimes,* 3=*about half of the time,* 4=*most of the time*, and 5= *always*. Each source of social support (that is guardians), classmates, teachers and peers) was included as a sub-scale in the analysis. | 7.45 | <0.001 |
| Social support from classmate | Continuous (5 items) |  | 5.46 | <0.001 |
| Social support from teachers | Continuous (6 items) |  | 7.72 | <0.001 |
| Social support from peers | Continuous (13 items) |  | 7.03 | <0.001 |
| School satisfaction | Continuous | School satisfaction items were adapted from the Multidimensional Students Life Satisfaction Scale (MSLSS).^6^ The school sub-scale of the MSLSS has demonstrated good reliability and validity in other contexts.^7,8^ Respondents were asked to rate 8 items on a 5- point scale, with 1=never, 2=almost never, 3=sometimes, 4=often and 5=almost always. The theoretical range for this scale is 8-40, with higher scores indicating higher levels of school satisfaction. The actual range was 17-40, with Cronbach’s alpha of 0.69 | 6.49 | <0.001 |
| Total family assets | Continuous | Family asset ownership was measured by a 21-item index that assessed ownership of tangible household assets. This index has been adapted from the demographic health surveys and has been tested globally.^9,10^Participants were asked, “Does the family you live in own the following: house, rentals, land, banana plantation, coffee plantation, car, bicycle, or television? The actual range for the sample in this study was 0-21, with Cronbach’s alpha of 0.72 | 1.55 | 0.121 |
| Primary care giver | Categorical | This indicates biological parents, grandparents, and other relatives | 5.58 | 0.061 |
| Food insecurity | Continuous | The Household Food Insecurity Access Scale (HFIAS) assessed respondents’ experiences of anxiety, uncertainty, insufficient quality, and insufficient food in their home in the last 12 months. The HFIAS has demonstrated strong psychometric properties in sub-Saharan Africa, including Tanzania. A validation study in rural Tanzania have confirmed its construct validity through factor analysis identifying domains of insufficient food quality and insufficient food intake, and have shown good internal consistency, with Cronbach’s alpha coefficients ranging from 0.83 to 0.90.^11^ The scale included 9 statements with 1-yes or 0-no responses. The actual range was 0-9, with a Cronbach’s alpha of 0.84 | -4.88 | <0.001 |
| Participants' personal savings | Continuous | Amount of monetary saving by the participant | 1.08 | 0.282 |
| Overall satisfaction with life | Ordinal | Participants were asked to rate on how they are satisfied with their life overall. Responses were rated on a 5- point Likert scale, with 1=not satisfied, 2=not very satisfied, 3= somewhat satisfied, 4=very satisfied, and 5= extremely satisfied. | 4.64 | <0.001 |
| Physical health | Ordinal | Participants were asked to rate their physical health is . Responses were rated on a 5- point Likert scale, with 1=very poor, 2=poor, 3= fair, 4=good, and 5= Excellent. | -3.37 | <0.001 |
| Low energy | Ordinal | Participants were asked to rate whether they have low energy. Responses were rated on a 5- point Likert scale, with 1=very poor, 2=poor, 3= fair, 4=good, and 5= Excellent. | 3.57 | <0.001 |
| Other medications taken by the participant | Categorical | Participants were asked whether they take any other medications, apart from the HIV medications prescribed to them (1= Yes, 0= No) | 0.24 | 0.626 |
| Number of different HIV medicines | Continuous | Participants were asked the number of different HIV medicines they normally have to take per day excluding Septrin | -2.06 | 0.04 |
| Self-efficacy in HIV treatment Adherence | Continuous | Self-efficacy in HIV treatment Adherence was assessed using a 12-item HIV Treatment Adherence Self-Efficacy Scale (HIV-ASES) which measured participants' levels of confidence to adhere to their HIV medicines in the past month.^12^ Responses were rated on 10-point Likert scale from 1=Cannot do it at all to 10= Certain I can do it. The theoretical ranged from 12-120 and the actual range was from 15 to 120. The HIV-ASES has demonstrated strong psychometric properties, including robust internal consistency (composite reliability coefficients = 0.90).^12^ In our sample, the scale demonstrated good internal reliability (Cronbach’s alpha =0.85). | -1.01 | 0.312 |
| intensive adherence counselling | Categorical | Participants were asked whether they have undergone intensive adherence counselling (1= yes or 0= No) | 7.67 | 0.006 |
| Barriers to Medical care | Continuous | Barriers to medical care were assessed by 10-items scale, participants were asked to agree =1 or disagree=0 on the different reasons why they did not get the HIV-related care they needed as recommended to them.^13^ Sample items included "I was unable to pay for medical care", "I did not have transportation to medical care", "I was treated poorly at a clinic in the past". The actual score ranged from 0 to 9 with higher scores indicating higher barriers to medical care. Similar adherence-barrier instruments have demonstrated acceptable internal consistency (Cronbach’s alpha ranging from 0.70 to 0.72)^14^. In the present study, the scale demonstrated acceptable internal reliability (Cronbach’s alpha = 0.75) | -4.95 | <0.001 |
| HIV shame | Continuous | HIV shame was measure by a 8-item Questionnaire adapted and validated among Ugandan youth living with HIV.^15^ Participants rated how true each statement was on a 3-point scale, with 0 =not true, 1 = somewhat true, and 2 =very true. The scale has demonstrated strong psychometric properties in Ugandan adolescents living with HIV, including good internal consistency (Cronbach’s alpha = 0.84) and criterion validity.^15^ The theoretical range is 0-16. The actual scores ranged from 0 to 16 with higher scores indicating higher levels of shame. In our sample, internal consistency was good (Cronbach’s alpha= 0.85). | -8.59 | <0.001 |
| Stigma by association at school | Continuous | Stigma by association at school was measured using 10-items adapted from the Brief Stigma-by Association Scale (BSAS).^16^ The scale measures experiences and consequences of associated stigma at the school environment, on a 3-point scale with 0= Not at all, 1= Sometimes and 2= All the time. The theoretical range for the scale is 0-20. Actual scores ranged from 0 to 20 with higher scores indicating higher levels of stigma by association at school. The BSAS demonstrated good internal consistency reliability (Cronbach’s alpha = 0.87) among African American urban adolescents.^16^ In our sample, the scale demonstrated good internal consistency (Cronbach’s alpha = 0.88). | -8.2 | <0.001 |
| HIV stigma | Continuous | Stigma was measured by the HIV Stigma Scale (HSS), a 40-item measure of stigma and psychosocial aspects of having HIV.^17^ Responses were captured using a 4-point scale with 1= strongly agree, 2= agree, 3=disagree and 4=strongly disagree. Items in the inverse direction were reverse coded to create summated scores, with higher scores indicating high levels of HIV-related stigma. The theoretical range for this scale is 40-160. Actual scores ranged from 43 to 154 with higher scores indicating higher levels of stigma. The HSS has demonstrated strong psychometric properties across diverse populations and settings. A recent systematic review reported that internal consistency estimates were acceptable to excellent (Cronbach’s alpha ≥ 0.70) in over 93% of studies, with many reporting alpha coefficients above 0.90.^18^ In our sample, the HSS demonstrated excellent internal consistency (Cronbach’s alpha = 0.93), indicating high reliability. | -4.34 | <0.001 |
| Hopelessness | Continuous | Hopelessness was measured using the Beck Hopelessness Scale. The 20-item scale measures children’s hopelessness and pessimistic attitudes toward the future. Items have a “true” or “false” response coded as “1” or “0” respectively.^19^ Items in the opposite direction were reversed-coded, with higher scores indicating higher levels of hopelessness. The actual scores ranged from 0 to 17 with higher scores indicating higher levels of hopelessness. The BHS has demonstrated strong psychometric properties across diverse populations. Standardization studies in general population samples have reported good internal consistency (Cronbach’s alpha ≈ 0.81).^20^ n our sample, the BHS demonstrated acceptable internal consistency (Cronbach’s alpha = 0.74). | -12.58 | <0.001 |
| Self-esteem | Continuous | Respondents’ self-esteem was measured using the Rosenberg Self-Esteem Scale.^21^ The 10-item scale measures individual self-esteem on a 4-point Likert- scale, with 4=*strongly agree*, 3=*agree*, 2=*disagree*, 1=*strongly disagree*. The theoretical range for the RSES is 10-40, with high scores indicating high self-esteem. Actual scores ranged from 11-40 with higher scores indicating higher levels of self-esteem. The RSES has demonstrated strong psychometric properties across diverse cultural contexts, including African populations. Validation studies have reported good internal consistency (Cronbach’s alpha typically ranging from 0.73 to 0.86).^22^ In the present sample, the RSES demonstrated good internal consistency (Cronbach’s alpha = 0.81) | 8.92 | <0.001 |
| Self-Concept | Continuous | Self-concept was measured using the Tennessee Self-Concept Scale (TSCS).^23^ The 20-item scale measures children’s perception of identity and self-satisfaction. Each of the 20 items was rated on a 5-point scale: 1= *always false,* 2=*usually false*, 3=*sometimes true/sometimes false*, 4=*usually true* and 5= *always true.* Ten (10) items in the opposite direction were reverse-coded to create summated scores. The theoretical range for the Tennessee Self-Concept Scale is 20-100. The actual scores ranged from 50 to 100 with higher scores indicating higher levels of self-concept. Validation studies of translated versions have reported good internal consistency (Cronbach’s alpha coefficients of 0.80).^24^ In the present sample, the TSCS demonstrated good internal consistency (Cronbach’s alpha = 0.81) | 11.04 | <0.001 |
| Viral loads suppression | Categorical | Study participants gave a blood sample to determine their HIV RNA viral loads. The collected blood was run for viral loads tests. We categorized viral loads into two categories that is HIV viral suppression and non-suppression. HIV viral suppression, defined as participants having HIV RNA viral loads of less than 1000 copies/ml based the World Health Organization guideline.^25^ | 0.025 | 0.874 |
| Poor antiretroviral therapy (ART) adherence | Continuous | Poor antiretroviral therapy (ART) adherence was assessed using six measures, including: the last time medication was missed, days medication was missed in the past 30 days, frequency of taking medication as prescribed, difficulty in adhering to medication, arguments with caregivers about medication, and frequency of missed doses in the past six months. The theoretical scores ranged from 0 to 6. Actual scores ranged from 0 to 6. | -4.53 | <0.001 |

**Supplementary Table 2: LASSO Coefficients**

|  | **Feature** | **Coefficient** |
| --- | --- | --- |
| **1** | Family care and relationships | -0.016914 |
| **2** | Social support by teacher | -0.017246 |
| **3** | Food insecurity | 0.009943 |
| **4** | ART Adherence self-efficacy | -0.00287 |
| **5** | HIV shame | 0.031086 |
| **6** | Stigma by association at school | 0.046694 |
| **7** | HIV stigma | 0.004725 |
| **8** | Hopelessness | 0.107214 |
| **9** | Self esteem | -0.022975 |
| **10** | Self-concept | -0.021438 |
| **11** | Self-reported poor ART adherence | 0.01899 |
| **12** | Orphanhood status | -0.006406 |

**Supplementary Table 3: Predictive performance of different ML models using Synthetic Minority Over-sampling Technique (SMOTE).**

| **Classifier** | **AUROC (mean (SD))** | **AUPRC (mean (SD))** | **Accuracy (mean (SD))** | **Precision (mean (SD))** | **Sensitivity (mean (SD))** | **Specificity (mean (SD))** | **F1-score (mean (SD))** |
| --- | --- | --- | --- | --- | --- | --- | --- |
| Random Forest | 0.76 (0.02) | 0.55 (0.04) | 0.66 (0.04) | 0.48 (0.04) | 0.83 (0.03) | 0.59 (0.07) | 0.60 (0.03) |
| Logistic Regression | 0.77 (0.05) | 0.59 (0.08) | 0.65 (0.08) | 0.47 (0.07) | 0.83 (0.02) | 0.57 (0.11) | 0.60 (0.06) |
| **SVM** | **0.77 (0.05)** | **0.61 (0.06)** | **0.65 (0.06)** | **0.47 (0.06)** | **0.82 (0.02)** | **0.58 (0.09)** | **0.60 (0.04)** |
| Gradient Boosting | 0.76 (0.02) | 0.56 (0.05) | 0.66 (0.05) | 0.47 (0.04) | 0.83 (0.03) | 0.58 (0.08) | 0.60 (0.03) |
| XGBoost | 0.74 (0.03) | 0.55 (0.04) | 0.64 (0.03) | 0.46 (0.03) | 0.82 (0.02) | 0.56 (0.05) | 0.59 (0.02) |
| Decision Tree | 0.70 (0.04) | 0.49 (0.09) | 0.54 (0.09) | 0.39 (0.05) | 0.86 (0.05) | 0.40 (0.14) | 0.54 (0.05) |
| LASSO | 0.77 (0.05) | 0.59 (0.08) | 0.67 (0.07) | 0.48 (0.07) | 0.83 (0.03) | 0.59 (0.09) | 0.61 (0.06) |

**Supplementary Table 4: Predictive performance of different ML models using Adaptive Synthetic Sampling (ADASYN)**

| **Classifier** | **AUROC (mean (SD))** | **AUPRC (mean (SD))** | **Accuracy (mean (SD))** | **Precision (mean (SD))** | **Sensitivity (mean (SD))** | **Specificity (mean (SD))** | **F1-score (mean (SD))** |
| --- | --- | --- | --- | --- | --- | --- | --- |
| Random Forest | 0.75 (0.03) | 0.54 (0.04) | 0.62 (0.06) | 0.45 (0.04) | 0.92 (0.00) | 0.46 (0.11) | 0.60 (0.04) |
| **Logistic Regression** | **0.77 (0.04)** | **0.60 (0.07)** | **0.59 (0.06)** | **0.43 (0.04)** | **0.92 (0.00)** | **0.42 (0.16)** | **0.58 (0.03)** |
| SVM | 0.46 (0.22) | 0.38 (0.20) | 0.39 (0.13) | 0.34 (0.06) | 0.94 (0.03) | 0.38 (0.19) | 0.49 (0.06) |
| Gradient Boosting | 0.74 (0.04) | 0.55 (0.05) | 0.57 (0.09) | 0.42 (0.05) | 0.92 (0.01) | 0.39 (0.17) | 0.57 (0.05) |
| XGBoost | 0.74 (0.04) | 0.54 (0.06) | 0.53 (0.05) | 0.39 (0.02) | 0.94 (0.04) | 0.39 (0.16) | 0.55 (0.03) |
| Decision Tree | 0.58 (0.06) | 0.47 (0.05) | 0.31 (0.01) | 0.31 (0.01) | 1.00 (0.00) | 0.41 (0.17) | 0.47 (0.01) |
| LASSO | 0.77 (0.04) | 0.60 (0.07) | 0.59 (0.05) | 0.42 (0.03) | 0.92 (0.00) | 0.43 (0.17) | 0.58 (0.03) |

**Supplementary Figure: Correlation matrix for model features.**


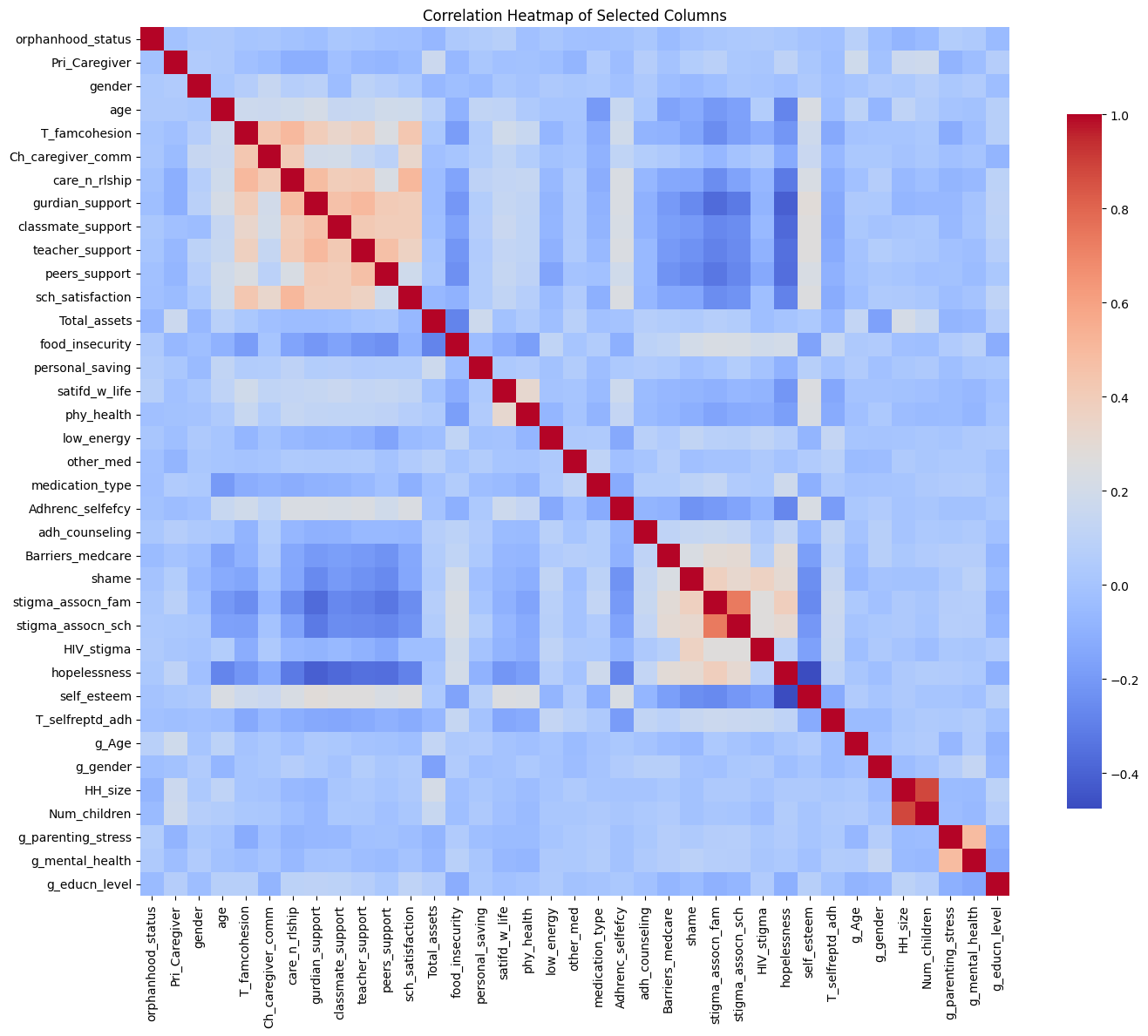


**Reference:**

1. Moos R, Moos B. *Family Environmental Scale Manual.* 3rd ed. Consulting Psychologists Press Inc.; 1994.

2. Skinner H, Steinhauer P, Santa-Barbara J. The Family Assessment Measure. *Can J Commun Ment Health*. 1983;2(2):91-103.

3. Vaux A, Riedel S, Stewart D. Modes of social support: The Social Support Behaviors (SS-B) Scale. *Am J Community Psychol*. 1987;15:209-237. doi:10.1007/BF00919279

4. Bukowski WM, Hoza B, Boivin M. Measuring Friendship Quality During Pre- and Early Adolescence: The Development and Psychometric Properties of the Friendship Qualities Scale. *J Soc Pers Relatsh*. 1994;11(3):471-484. doi:https://doi.org/10.1177/0265407594113011

5. Erkan Ati̇K Z, Çok F, Esen Çoban A, Doğan T, Güney Karaman N. The Turkish Adaptation of the Friendship Qualities Scale: A Validity and Reliability Study. *Educ Sci Theory Pract*. Published online April 28, 2014. doi:10.12738/estp.2014.2.1778

6. Huebner ES. Manual for the multidimensional students’ life satisfaction scale. *Dep Psychol Univ S C*. Published online 2001.

7. Bradley KD, Cunningham JD, Gilman R. Measuring Adolescent Life Satisfaction: A Psychometric Investigation of the Multidimensional Students’ Life Satisfaction Scale (MSLSS). *J Happiness Stud*. 2014;15(6):1333-1345. doi:10.1007/s10902-013-9478-z

8. Schnettler B, Orellana L, Sepúlveda J, et al. Psychometric properties of the Multidimensional Students’ Life Satisfaction Scale in a sample of Chilean university students. *Suma Psicológica*. 2017;24(2):97-106. doi:10.1016/j.sumpsi.2017.06.001

9. Moser C, Felton A. *The Construction of an Asset Index." Poverty Dynamics: Interdisciplinary Perspectives*. Vol 10. 2009.

10. Naveed TA, Gordon D, Ullah S, Zhang M. The Construction of an Asset Index at Household Level and Measurement of Economic Disparities in Punjab (Pakistan) by using MICS-Micro Data. *Soc Indic Res*. 2021;155(1):73-95. doi:10.1007/s11205-020-02594-3

11. Knueppel D, Demment M, Kaiser L. Validation of the Household Food Insecurity Access Scale in rural Tanzania. *Public Health Nutr*. 2010;13(3):360-367. doi:10.1017/S1368980009991121

12. Johnson MO, Neilands TB, Dilworth SE, Morin SF, Remien RH, Chesney MA. The Role of Self-Efficacy in HIV Treatment Adherence: Validation of the HIV Treatment Adherence Self-Efficacy Scale (HIV-ASES). *J Behav Med*. 2007;30(5):359-370. doi:10.1007/s10865-007-9118-3

13. Kalichman SC, Catz S, Ramachandran B. BARRIERS TO HIV/AIDS TREATMENT AND TREATMENT ADHERENCE AMONG AFRICAN-AMERICAN ADULTS WITH DISADVANTAGED EDUCATION.

14. Mueller S, Wilke T, Gorasso V, Erhart M, Kittner JM. Adaption and validation of the adherence barriers questionnaire for HIV patients on antiretroviral therapy (ABQ-HIV). *BMC Infect Dis*. 2018;18(1):599. doi:10.1186/s12879-018-3530-x

15. Michalopoulos LM, Meinhart M, Barton SM, et al. Adaptation and Validation of the Shame Questionnaire Among Ugandan Youth Living with HIV. *Child Indic Res*. 2019;12(3):1023-1042. doi:10.1007/s12187-018-9570-3

16. Mason S, Berger B, Ferrans CE, Sultzman V, Fendrich M. Developing a Measure of Stigma by Association With African American Adolescents Whose Mothers Have HIV. *Res Soc Work Pract*. 2010;20(1):65-73. doi:10.1177/1049731508330223

17. Berger BE, Ferrans CE, Lashley FR. Measuring stigma in people with HIV: Psychometric assessment of the HIV stigma scale¶. *Res Nurs Health*. 2001;24(6):518-529. doi:10.1002/nur.10011

18. Wanjala SW, Too EK, Luchters S, Abubakar A. Psychometric Properties of the Berger HIV Stigma Scale: A Systematic Review. *Int J Environ Res Public Health*. 2021;18(24):13074. doi:10.3390/ijerph182413074

19. Beck AT, Weissman A, Lester D, Trexler L. The measurement of pessimism: The Hopelessness Scale. *J Consult Clin Psychol*. 1974;42(6):861-865. doi:10.1037/h0037562

20. Kocalevent RD, Finck C, Pérez-Trujillo M, Sautier L, Zill J, Hinz A. Standardization of the Beck Hopelessness Scale in the general population. *J Ment Health*. 2017;26(6):516-522. doi:10.1080/09638237.2016.1244717

21. Rosenberg M. Society and the adolescent self-image. Published online 1995.

22. Makhubela M, Mashegoane S. Psychological validation of the Rosenberg Self-Esteem Scale (RSES) in South Africa: Method effects and dimensionality in black African and white university students. *J Psychol Afr*. 2017;27(3):277-281. doi:10.1080/14330237.2017.1294303

23. Fitts WH, Warren WL. Tennessee self-concept scale: TSCS-2. Published online 1996.

24. Fernández-Zabala A, Rodríguez-Fernández A, Goñi A. The structure of the Social Self-Concept (SSC) Questionnaire. *An Psicol*. 2015;32(1):199. doi:10.6018/analesps.32.1.193931

25. WHO. The role of HIV viral suppression in improving individual health and reducing transmission. Published online July 22, 2023. Accessed June 18, 2024. https://www.who.int/publications/i/item/9789240055179
